# Supplementary material for: High-efficiency green management of potato late blight by a self-assembled multicomponent nano-bioprotectant
Source: Nat Commun. 2023 Sep 12;14:5622. doi: 10.1038/s41467-023-41447-8 (PMC10497615; doi:10.1038/s41467-023-41447-8)
Supplement: Supplementary file 1 — Supplementary Information [file 41467_2023_41447_MOESM1_ESM.pdf]

High-efficiency green management of potato late blight by a self-assembled multicomponent nano-bioprotectant

Wang *et al.*

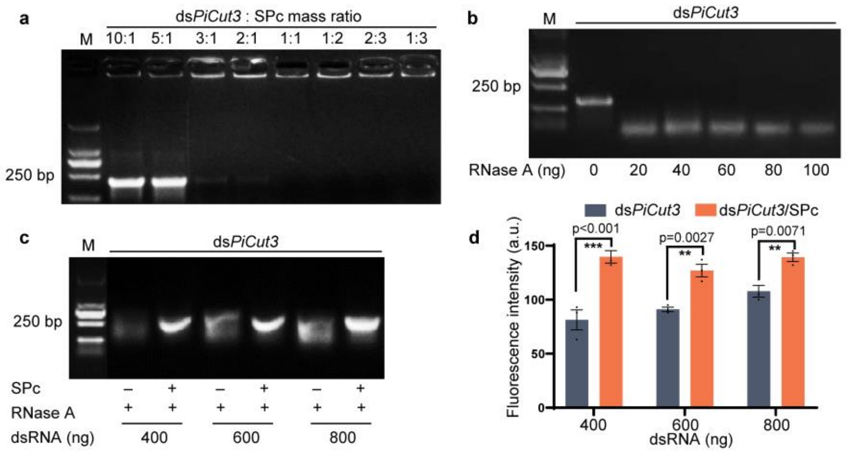

Supplementary Figure 1. Enhanced stability of SPc-loaded dsRNA. a, Gel electrophoresis assay of ds*PiCut3* retardation by SPc. 500 ng ds*PiCut3* was mixed with SPc at various mass ratios, and the mixture (8 μL) was incubated and then analyzed. M: marker. Each treatment was repeated three times. **b,** ds*PiCut3* degradation by RNase A. One μg ds*PiCut3* was mixed with RNase A to prepare the reaction solution, and the mixture was incubated for 20 min at 37°C. Each treatment was repeated three times. **c, d,** Gel electrophoresis assay (**c**) and relative band density (**d**) of SPc-loaded ds*PiCut3* treated with RNase A. RNase A was used to treat the ds*PiCut3*/SPc complex. Then the ds*PiCut3*/SPc complex was decomplexed in 0.3% SDS solution. Each treatment was repeated three times. Analyzed by two-way ANOVA with Tukey's HSD multiple comparison post hoc test (***p* < 0.01 and *** *p* < 0.001). Bars represent the mean ± SE. Source data are provided as a Source Data file.

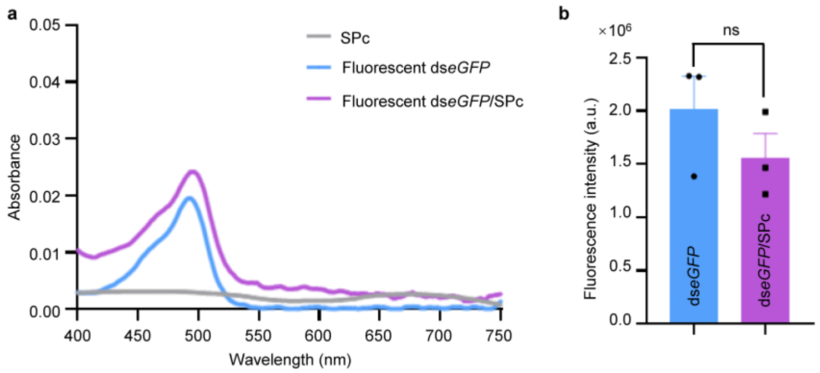

Supplementary Figure 2. Fluorescence intensity of ds*eGFP* assembled with SPc. a. The maximum absorptions of both fluorescent ds*eGFP* and ds*eGFP*/SPc complex were observed at 488-500 nm. The fluorescent ds*eGFP* was mixed with SPc at the mass ratio of 1:1 (final concentration of ds*eGFP*: 20 ng/μL). **b.** The fluorescence intensity of ds*eGFP* and ds*eGFP*/SPc complex. The comparison was conducted using independent *t*-test at the $p < 0.05$ level of significance. Source data are provided as a Source Data file.

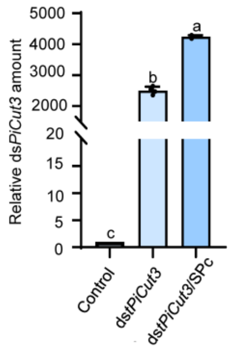

Supplementary Figure 3. Higher relative amount of SPc-loaded ds*PiCut3* on plant leaves. Bars represent the mean ± SE. Different letters above each bar indicate significant differences at $p < 0.05$ as determined by Tukey's HSD test. Source data are provided as a Source Data file.

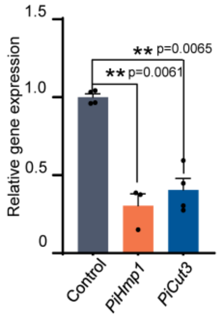

Supplementary Figure 4. Gene silencing efficiency of ds*PiHmp1*+*PiCut3* complex without SPc loaded *in vitro*. A mixture of *P. infestans* hyphae and spores (12 days) was collected and treated by ds*PiHmp1*+*PiCut3* complex without SPc loaded in vitro or ddH_2O as a control respectively. Gene expression of the above mixture was examined at 12 h after the treatment by qPCR. Analyzed by two-way ANOVA with Tukey's HSD multiple comparison post hoc test (**p < 0.01). Bars represent the mean ± SE. Source data are provided as a Source Data file.

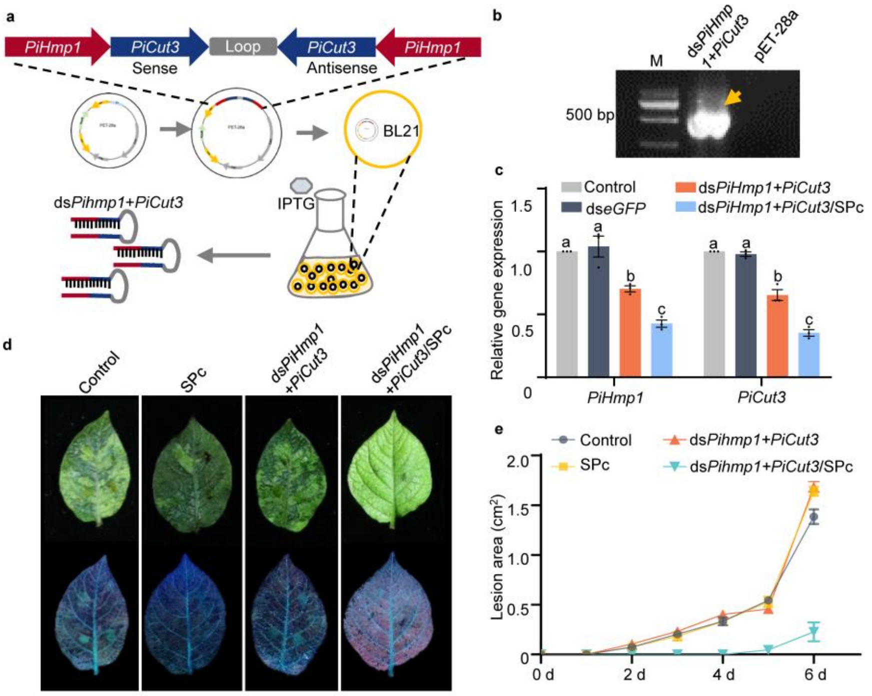

Supplementary Figure 5. Construction of dsPiHmp1+PiCut3/SPc complex and its protection assay. a, Reverse complementary fragments from *PiHmp1* and *PiCut3* were tandemly constructed into the pET28a expression vector, transformed into BL21 (DE3) RNaseIII- strain and then lysed by lysozyme to release ds*PiHmp1*+*PiCut3*. **b**, dsRNA expressed in pET28-BL21 (DE3) RNase III- *E. coli* expression system. Yellow arrow indicates the target dsRNA. M: marker. **c,** Gene silencing efficiency of ds*PiHmp1*+*PiCut3* and ds*PiHmp1*+*PiCut3*/SPc complex. A mixture of *P. infestans* hyphae and spores (12 days) was collected at 24 h after the treatment of ds*PiHmp1*+*PiCut3*. Different letters above each bar indicate significant differences at $p < 0.05$ as determined by Tukey's HSD test. **d, e,** Enhanced protective effect of SPc-loaded ds*PiHmp1*+*PiCut3* toward potato leaves. Various formulations were sprayed onto leaves, and sporangia suspension was inoculated to plant leaves after 24 h. Pictures were acquired at 6 dpi, and then lesion area was measured (n=3 biologically independent replicates). Bars represent the mean ± SE. Source data are provided as a Source Data file.

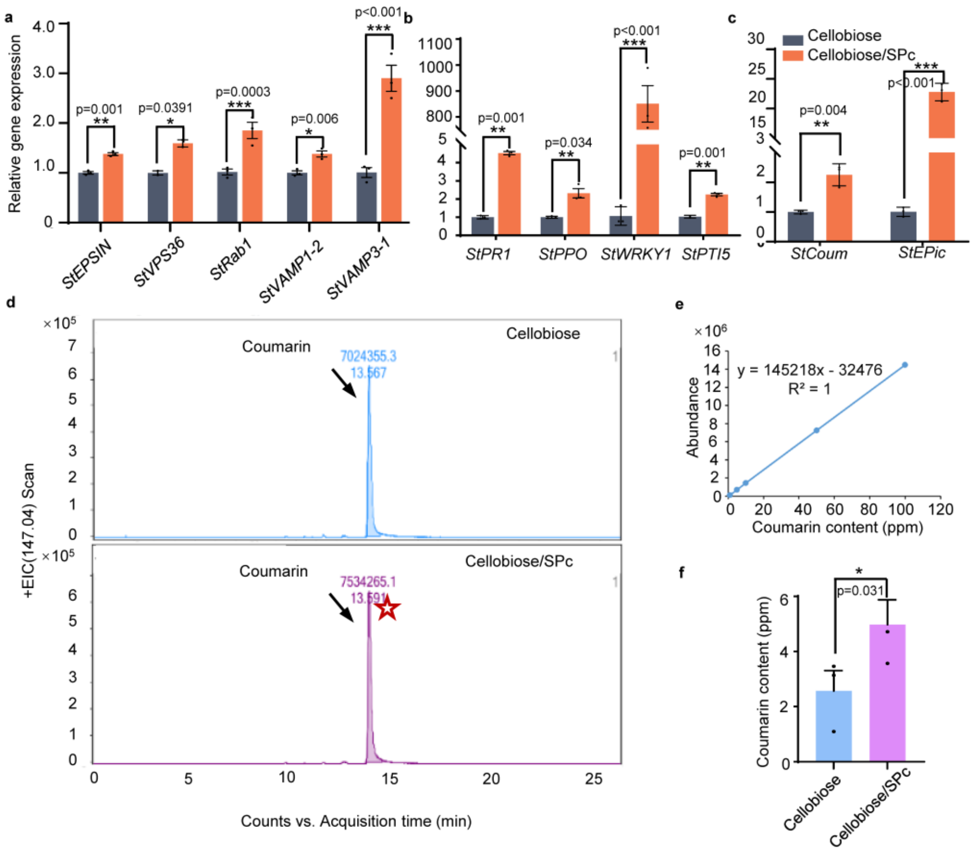

Supplementary Figure 6. Elevated bioactivity of cellobiose with the aid of SPc. a-c, SPc-loaded cellobiose up-regulated the expression of endocytosis-related genes, (**a**), cellobiose-induced immunity genes, (**b**) and phytoalexin-related genes, (**c**) in potato leaves compared with cellobiose alone. Each treatment was repeated three times. (**a**-**c**, Analyzed by two-way ANOVA with Tukey's HSD multiple comparison post hoc test) (*$p < 0.1$, **$p < 0.01$, ***$p < 0.001$). **d-f,** SPc-loaded cellobiose increased the coumarin content in potato leaves compared with cellobiose alone. (**d**) Chromatogram of coumarin. (**e**) Standard curve of coumarin for LC-MS/MS and (**f**) statistics of coumarin content. Bars represent the mean ± SE. Statistical analysis was conducted using independent two-tailed *t*-test (*$p < 0.05$, **$p < 0.01$ and ***$p < 0.001$). Source data are provided as a Source Data file.

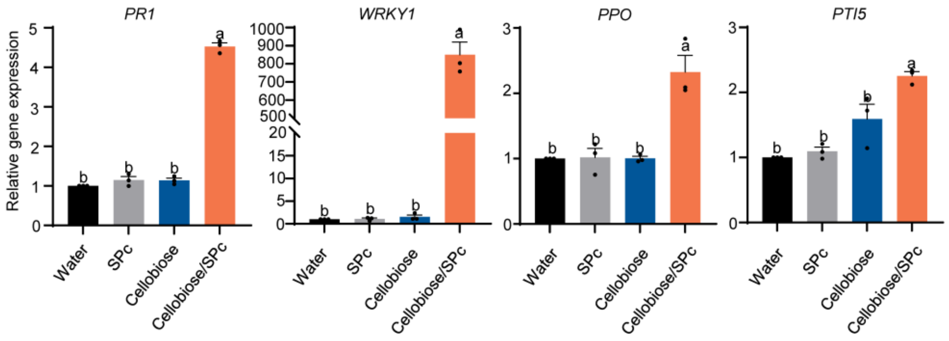

Supplementary Figure 7. SPc exhibited no influence on the expression of plant immunity genes. Bars represent the mean ± SE. Different letters above each bar indicate significant differences at $p < 0.05$ as determined by Tukey's HSD test. Each treatment was repeated three times. Source data are provided as a Source Data file.

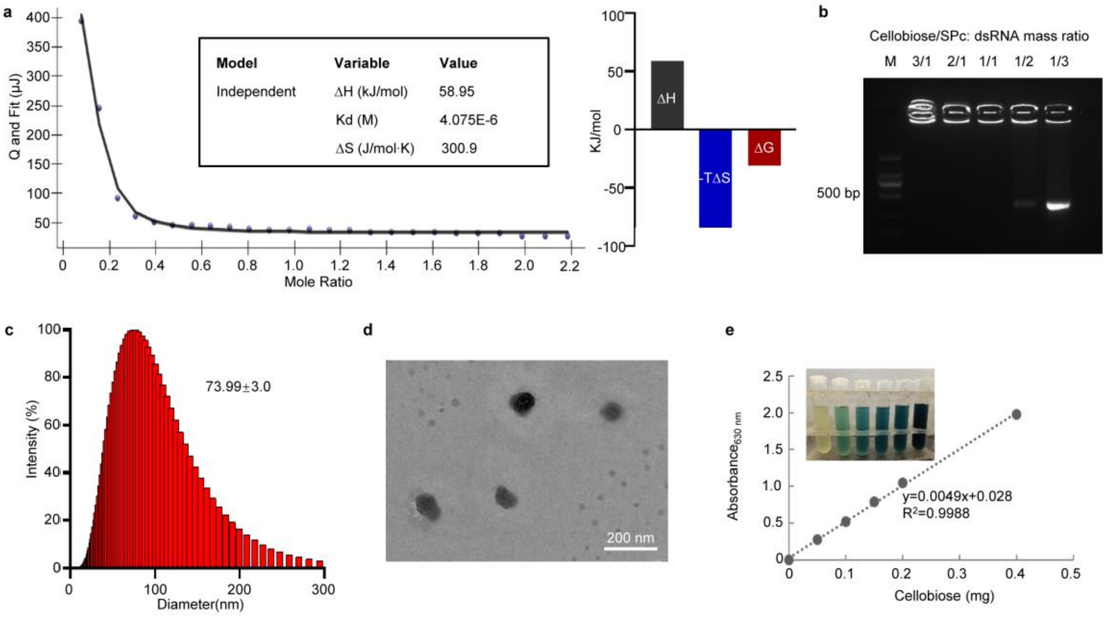

Supplementary Figure 8. Self-assembly mechanism of cellobiose/SPc complex and component quantification of cellobiose/SPc/dsRNA complex. a, ITC titration of cellobiose (0.138×10^{-3} mol/L) into SPc solution (0.1×10^{-3} mol/L). **b,** Gel electrophoresis assay of dsRNA retardation by cellobiose/SPc complex at various mass ratios. M: marker. **c.** The particle size distribution of SPc. The number indicates the average particle size. **d.** TEM image of SPc. **e,** Standard curve of cellobiose for anthrone-sulfuric acid colorimetry. n=3 biological replicates. Source data are provided as a Source Data file.

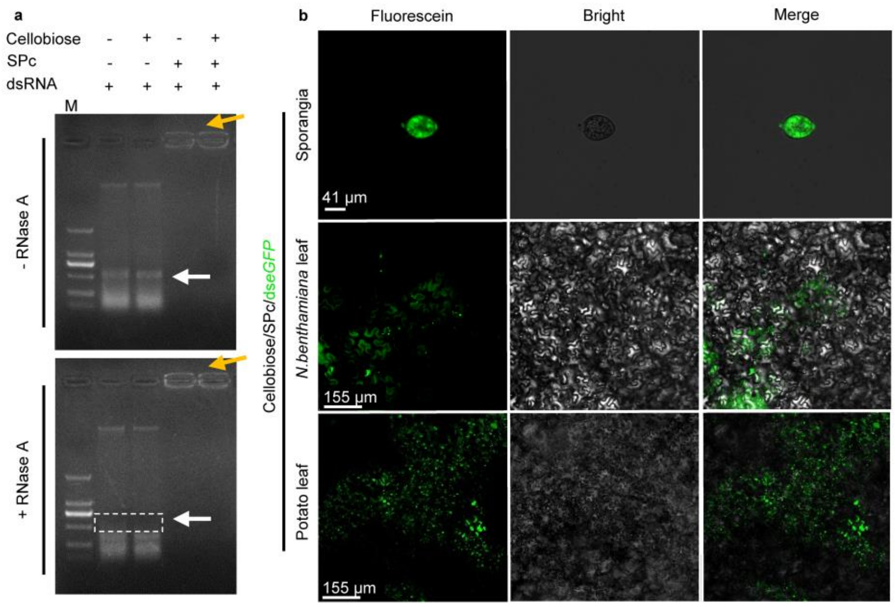

Supplementary Figure 9. The multicomponent nano-bioprotectant protects and delivers dsRNA to cells. a, Gel electrophoresis assay of multicomponent nano-bioprotectant treated with RNase A (100 ng). White arrow indicates the target dsRNA. Yellow arrow indicates the multicomponent nano-bioprotectant. M: marker. **b,** The multicomponent nano-bioprotectant can also be taken up by *P. infestans* sporangia, *N. benthamiana* and potato leaves. Multicomponent nano-bioprotectant was added to *P. infestans* sporangia (12 d), and fluorescence intensity was measured at 12 h after the application (n=3 biological replicates). Source data are provided as a Source Data file.

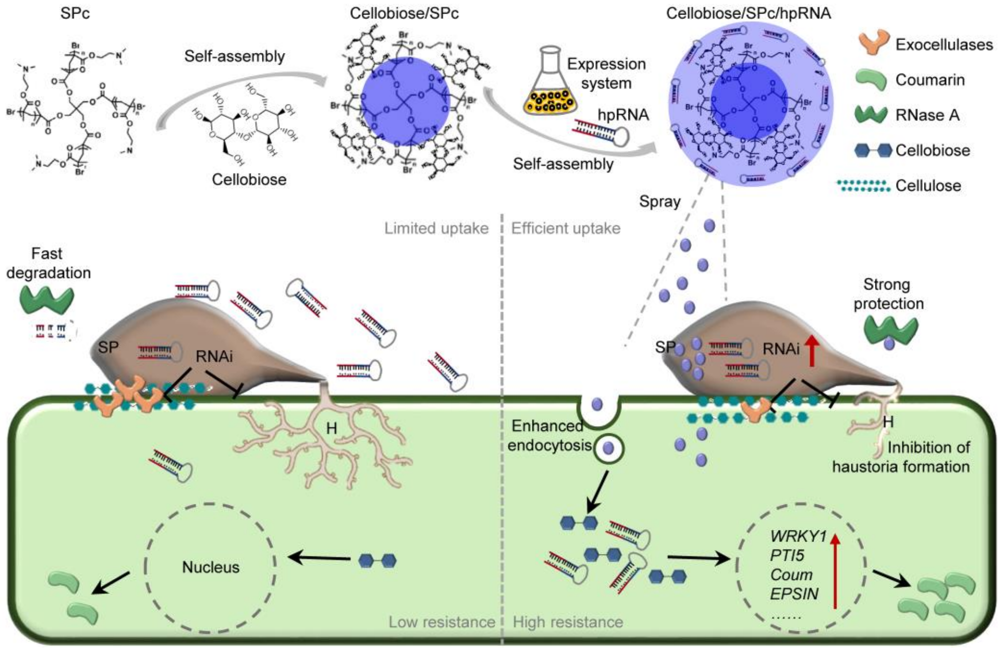

Supplementary Figure 10. Schematic illustration of multicomponent nano-bioprotectant preparation and application. SPc protects dsRNA from degradation by RNase A and increases dsRNA uptake by oomycetes and plants for highly-efficient dsRNA delivery. SPc decreases the particle size of the plant elicitor down to nanoscale, dramatically amplifying the plant defense responses against pathogens. SPc self-assembled with cellobiose and dsRNA to form a multicomponent nano-bioprotectant that is applied to control potato late blight. The current study realizes the sustainable green management of potato late blight via two aspects pathogen inhibition and plant defense. SP: sporangium; H: haustoria.

Supplementary Table 1. Loading capacity of SPc toward cellobiose.

Cellobiose (mg)	SPc (mg)	Cellobiose/SPc (mg)	Drug loading content	Average drug loading content[a]
100	100	114.5	12.66%	
100	100	116	13.79%	12.70±1.07%
100	100	113.2	11.66%	

[a] Mean ± SE.

Supplementary Table 2. Primers used in the current study.

Application	Primer name	Primer sequence (5'~3')	Accession number
PCR amplification for *P. infestans* genes to be essential for pathogenesis	*PiHmp1*-F	TTGTTGACCAGCTCGTTGAG	XM_002908934.1
	PiHmp1-R	CCATCACCCTTCTTCTTCCA	
	PiCut3-F	CAACCACGTCGTGTCTATCG	XM_002900240.1
	Picut3-R	GTTGCAGAACTCAATGGCCT	
Template DNA for *PiHmp1- PiCut3* synthesis *in vitro*	*PiHmp1*-dsRNA-T7-F	GTAATACGACTCACTATAGGTTGTTGACCAGCTCGTTGAG	
	PiHmp1-dsRNA-T7-F	GTAATACGACTCACTATAGGCCATCACCCTTCTTCTTCCA	
	PiCut3-dsRNA-T7-F	GTAATACGACTCACTATAGGCAACCACGTCGTGTCTATCG	
	PiCut3-dsRNA-T7-R	GTAATACGACTCACTATAGGGTTGCAGAACTCAATGGCCT	
Template DNA for *tdTomato* and *eGFP* synthesis *in vitro*	*tdTomato*- dsRNA-T7-F	GTAATACGACTCACTATAGGCAACATGGCCGTCATCAAAGA	
	tdTomato -dsRNA-	GTAATACGACTCACTATAGGCTTGTACAGCTCGTCCATGCC	
	eGFP-dsRNA-T7-F	TAATACGACTCACTATAGGCACAAGTTCAGCGTGTCCG	
	eGFP-dsRNA-T7-R	TAATACGACTCACTATAGGGTTCACCTTGATGCCGTTC	
Overlap PCR for constructing pET28a-*PiHmp1-PiCut3* vector	ovhmcut3-1 ZF	CTCGAGATGCCGATATGGGTAAGCA	
	ovhmcut3-1 ZR	ACGAATACCGGCCATCACCCTTCTTCTTCCAC	
	ovhmcut3-2 ZF	GGGTGATGGCCGGTATTCGTCCTTGGAGGGT	
	ovhmcut3-2 ZR	AAGCTTCTCGGCCGCG	
	ovcut3hm-1 reF	AAGCTTGTTGCAGAACTCAATGGC	
	ovcut3hm-1 reR	GGGTGATGGCCGGTATTCGTCCTTGGAGGGT	
	ovcut3hm-2 reF	ACGAATACCGGCCATCACCCTTCTTCTTCCAC	
	ovcut3hm-2 reR	TCTAGACCGATATGGGTAAGCATGT	
qRT-PCR for phytoalexin gene expression	*StCoum*-qRT-F	CGCCTCACAATCTCACACTC	OL412004.1
	StCoum-qRT-R	CGCATCTGATAGTAGGTCCGT	
	StEpic-qRT-F	AGCCGGTTCCTAATGCCTTC	XM_006349390.2
	StEpic-qRT-R	CGTTTTGTGCGTATCAGGGTC	
qRT-PCR for	*StEPSIN*-qRT-F	TGTCTTTGCTCCTTCCTTTACAC	XM_006349904.1

endocytosis gene expression	*StEPSIN*-qRT-R	TTCACCCCTCTCTTGAGGTC	
	StVPS36-qRT-F	CAAGAATCCACCACCTCCCC	XM_006347647.2
	StVPS36-qRT-R	AATCACCACGACCCCTTTCG	
	StRab-qRT-F	TGGTGAATGTCACTGCGCTA	CP055242.1
	StRab-qRT-R	CGATTTCAACCTGTTCGCGT	
	StVAMP1-2-qRT-F	GAAGGCTCAGGTTTCAGAAG	XM_006364202.2
	StVAMP1-2-qRT-R	GTGACACTTGAAACCACCA	
	StVAMP3-1-qRT-F	GGGAACGGTGATTTTGGCTG	XM_049539029.1
	StVAMP3-1-qRT-R	CGCAATGGGAAGTTGTCTGC	
qRT-PCR for immune gene expression	*StPR1*-qRT-F	ATGTGGGACGATGAGAAGCA	XM_006367028.1
	StPR1-qRT-R	CAAAGGCCGGTTGTTGATCT	
	StWRKY1-qRT-F	GAAGAATAAAGCCGGGTTCTTGG	NM_001288675.1
	StWRKY1-qRT-R	CAGTTTCATGGGCAGATCATCG	
	StPPO-qRT-F	GACACGAAGAAGATGGGATACG	U22921.1
	StPPO-qRT-R	TCGAGTTTAGCCAATGGGAAT	
	StPTI5-qRT-F	TATTCTTCGTCCATTACAG	XM_006367134.2
	StPTI5-qRT-R	AACAGAGGCGTTCACTA	
qRT-PCR for *tdTomato* gene	*PitdTomato*-qRT-F	CAACAACATGGCCGTCATCAAA	
	PitdTomato-qRT-R	TGTACAGCTCGTCCATGCCG	
qRT-PCR for reference *actin* gene expression	*StEF1a*--qRT-F	ATTGGAAACGGATATGCTCCA	XM_022311019.1
	StEF1a-qRT-R	TCCTTACCTGAACGCCTGTCA	
	PiActin-qRT-F	CAATTCGCCACCTTCTTCGA	XM_002897714.1
	PiActin-qRT-R	GCCTTCCTGCCCTCAAGAAC	

Supplementary Table 3. Potato disease classification.

Classification	Description
0	No lesion area
1	The lesion area accounted for less than 5% of the whole leaf area
3	The lesion area accounted for 6% ~ 10% of the whole leaf area
5	The lesion area accounted for 11% ~ 20% of the whole leaf area
7	The lesion area accounted for 21% ~ 50% of the whole leaf area
9	The lesion area accounted for more than 50% of the whole leaf area
